# Impression Making

**Published:** 1912-07-15

**Authors:** N. S. Hoff


### IMPRESSION MAKING.

BY N. S. HOFF, D.D.S.

Read before the Northern Michigan Dental Society, June 6, 1912.

The three impression materials, wax, modeling compound and plaster of Paris, each have peculiarities which can be utilized advantageoulsy in the different conditions of the alveolar and oral structures. Bees wax, because of its tenacity and ease with which it can be softened is useful very largely in taking impressions when there are hard and soft areas to be included in the impression. Modeling compound is similarly useful, but because of its tendency to flow while warm under pressure, it has no such condensing quality as the wax. Plaster of Paris has no condensing quality unless used so near its setting point, that its most characteristic quality of taking detail is lost.

The object of this paper and clinic is to show a method of combin'ng the best qualities of bees-wax and plaster to obtain true form and accurate detail in impression taking. The method is not new, but I want to call attention to the details of manipulation which are essential to insure the highest type of success. If the details of the method are not carefully and completely followed, the results will be more or less problematic. The method can be used in all edentulous cases, but it is especially useful in cases where patients have been wearing badly fitting dentures, or dentures badly occluding with the antagonizing teeth, with the result that much soft or flabby gum tissue exists. It is necessary in order to get an equally firm foundation for supporting the denture, that this soft or loose tissue be condensed or pushed aside by the impression material until there is an equally resistant foundation under the impression such as will be produced when the force of mastication is applied to the completed denture. The claim is made by some operators that all this can be accomplished with modeling compound used alone. But when we remember the high heat necessary to soften the compound and its tendency to flow under some slight pressure when soft, we can readily believe that it has no adequate displacing function unless used so hard that it will not take either the form or detail of a mouth having many undercuts. It can be manipulated by great care in many cases with desirable results, but it requires so much care and skill that few operators will have the patience to use in those cases where accurate results are essential. The method we describe is so much easier and simpler that it seems preferable in all difficult cases.

The tray used should neither be too large nor too small, nor should it be badly adapted. One of the worst faults most dentists have, is that they do not provide themselves with a sufficient number or variety of impression trays, and they use these long after their usefulness has expired. The tray should be from an eighth to three sixteenth of an inch larger than the alveolar process so as to have a sufficient thickness of wax over the process, that the wax will not be bent out of form when withdrawing it from the mouth. If the tray does not properly conform to the mouth, bend or trim it until it fits approximately. This is a first important step, as a uniform layer of wax, and that only in the necessary quantity, will produce better results than a great mass in a poorly adapted tray.

Warm the wax in hot water, being careful not to make it granular by over-heating and too much manipulation. Have the tray dry and slightly warm; then quickly kneed the wax in the palm of the hand to the general form of the tray, and as quickly place it in the tray in the form to take the impression with the least amount of flowing of the wax in the tray. Insert the tray with the wax into the mouth, immediately after passing its surface over the heat from a bunsen flame, being careful not to melt it or have it too hot for the tender mouth. Carefully adjust and force it only partly to place when it should be removed and inspected to determine whether the tray was properly fitted and the wax properly placed. If there is an excess of wax in one part of the tray, cut it out with a warm knife and if there is a lack in some other part add what may be needed. This can all be done in less time than it takes to tell it, and the wax will not have chilled so much but that the impression, after passing its surface over the bunsen flame to slightly resoften it, can be reinserted and forced almost to its final position. While holding it firmly in contact, ask the patient to try and dislodge it with his lips and tongue. This will have the effect of distinctly outlining the alvevolar margins and give you the probable limits of the plate when completed. It will also give in distinct outline the markings of the muscle tendons which prevent close sealing of the plate margins. After the patient has made this effort, have him relax, and then force the wax impressiou *hard* against the tissues. By this time the wax has cooled enough so that it will not flow except under great pressure, but it will condense the soft tissues while the hard parts of the mouth will be impressed into the wax. If all these steps have been carefully taken the impression will adhere so tenaciously, that it cannot easily be dislodged except by lateral movements, or the admission of air under its borders.

Now remove the impression from the mouth, and with a warm knife trim the impression margins to a point only slightly beyond the intended limits of the plate rim, and also the palatal portion to extend about an eighth of an inch beyond the plate limits. Cut away at the fraeni and buccal muscle attachments, but extend high in the cuspid regions, and also well up on the tuberosities of the upper jaw and down on the lingual surfaces in the third molar regions.

Now make an exploratory examination of the mouth and note the points where the gum is dense and firm or unyielding to pressure. The wax impression in these corresponding regions should be carved out to relieve the pressure on the hard spots and to throw it onto the soft areas. The depth and extent of this carving will depend on the particular case, and one will soon acquire the judgment necessary to secure proper results. Generally the hard areas of the upper jaw are in the median line of the vault extending from the soft palate to the incisor teeth, and on the top of the alveolar ridge in the bicuspids and molar locations. A groove made with a warm carver or spatula from the cuspids to the last molar will usually be indicative. In making this groove do not allow it to approach too closely to the buccal surfaces of the alveolus, as we want pressure there to secure a tight sealing of the plate rim margins. Do not carve in the region of the soft palate or between the median ridge and the alveolar process in the molar region, as this area is almost always soft. The region of the rugae just back of the incisal region and the intermaxillary ridge should be carved away to prevent compression of these lines.

In the lower jaw the carving is uaually indicated mostly on the top of the alveolar ridge all the way around from one end of the plate to the other. After the wax impressions are carved their surfaces should be slightly warmed and placed in the mouth and forced up to place. If the carving has been properly done, there will be such a suction that the impression will be removed only with difficulty.

So far the process has resulted only in giving us the general form in the condition that we want it for the plate. We will now mix a small amount of fine impression plaster, using potassium sulphate to make it set quickly, and place this in the wax impression so that it covers the entire surface. This is quickly inserted into the mouth before it begins to set and the impression forced into place and held there, while the patient is asked to draw the lips and cheek muscles down tight over the margins of the impressions to get distinct marking on the impression margins. This they will do quickly and then relax. The operator will immediately force the impression *hard* to place and hold it firmly until the plaster has set. It will be found that the impression so taken will adhere so tenaciously that it is difficult to remove, and when it is removed, its surface will show in detail every line of the surface of the mouth.

An impression made in this way will certainly produce a more accurate form and more detail than can be had by any one material and we believe by any other combination of impression materials. And models made from such impressions ought to produce well adapted dentures. The method is not so well adapted to taking partial impressions, and very deep undercuts will bother some as the wax will not break as plaster alone will do. The value of the method depends wholly upon the exactness of its manipulation. The reason I present it to you is not that it is new in method, but because it is the most definite and satisfactory method of securing impressions in the kind of cases that cause us the most trouble in fitting dentures for chronic cases. I want however, to insist that the details so outlined be studiously followed, as careless manipulation at any stage of the procedure may result in failure. We should also remember, however, that a good impression does not always mean that the denture will be all that we expect. The other procedures are just as important, and scientific accuracy only secures results.

